# MiniMizer Gastric Ring displacement at 31 weeks of gestation as a life-threatening complication

**DOI:** 10.1007/s11695-024-07521-w

**Published:** 2024-10-05

**Authors:** Paulina Szymczak, Magdalena Emilia Grzybowska, Adam Grzeczka, Michał Szymański, Dariusz Grzegorz Wydra

**Affiliations:** 1 Department of Gynecology, Obstetrics and Neonatology, Medical University of Gdansk, Gdańsk, Poland; 2 University Clinical Centre in Gdansk, Gdańsk, Poland; 3 Department of Surgical Oncology, Transplant Surgery and General Surgery, Medical University of Gdansk, Gdańsk, Poland

**Keywords:** Bariatric surgery, Minimizer ring, Pregnancy, Complications

## Abstract

This case report describes a pregnant woman who underwent a laparoscopic MiniMizer Gastric Ring procedure for clinically severe obesity only one month before conception. At 31 weeks of gestation, the patient as admitted to the hospital with postprandial vomiting and persistent left-sided colicky abdominal pain. Maternal abdominal MRI revealed an intestinal obstruction and elective surgery was recommended. Due to the considerable risk to the fetus, antenatal corticosteroids were immediately administered to promote lung maturation and magnesium sulfate was started for fetal neuroprotection. During an exploratory laparoscopy, significantly enlarged and ischemic intestinal loops were found, leading to the decision to perform an atraumatic “en caul” cesarean delivery. After a successful “en caul” delivery, the MiniMizer ring, which had dislodged downwards and led to mesenteric ischemia, was visualized. Intraoperative esophagogastroduodenoscopy revealed a 1cm defect in the stomach wall related to gastric ring, covered with purulent exudate. Further exploration, showed a herniation of the distal alimentary loop through the Petersen foramen. Successful management included ring removal and intestinal loop reduction from the Petersen’s space, without evidence of strangulation, as confirmed with indocyanine green (ICG) angiography. The postoperative course was uneventful. Women with obesity who have undergone bariatric surgery should to be informed of the increased likelihood of becoming pregnant after treatment. It is advised to notify the patient of the importance of maintaining a sufficient interval between bariatric surgery and conception. Additionally reports from the literature on various complications during pregnancy after bariatric surgery are presented.

## Case Description

A 39-year-old woman (gravida 2, para 1, history of cesarean section) was admitted to a tertiary referral hospital at 31 weeks of gestation due to postprandial vomiting and persistent, left-sided colicky abdominal pain. Due to clinically severe obesity, the patient had undergone a One Anastomosis Gastric Bypass (OAGB) 12 years previously and also a laparoscopic MiniMizer Gastric Ring procedure, only one month before conception. At 4 months of gestation, the patient developed recurrent vomiting, which increased in intensity and resulted in restricted intake of solid foods. Maternal BMI (body mass index) upon admission was 34 kg/m2. During the current pregnancy, the patient experienced a weight reduction of 11 kg, which did not negatively affect the fetus.

## Methods and Results

**Fig. 1:**
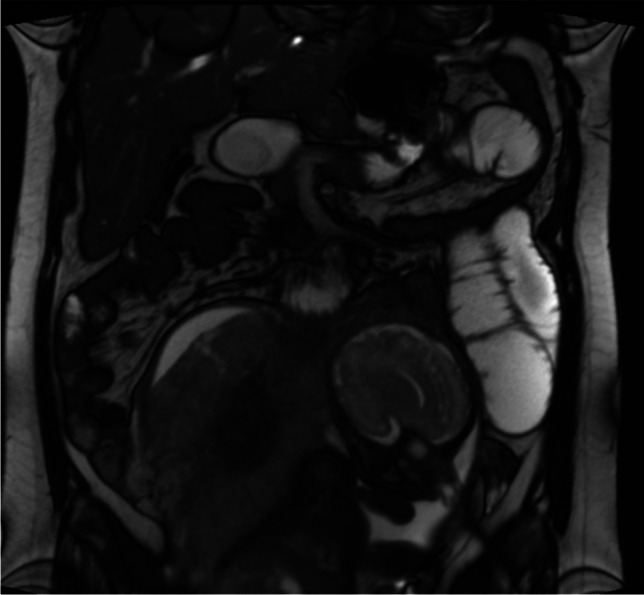
Intestinal obstruction visualized on MRI

Magnetic resonance imaging (MRI) of the maternal abdomen revealed an intestinal obstruction [Fig. 1] and elective surgery was advised. An exploratory laparoscopy found significantly enlarged and ischemic intestinal loops. In light of the considerable risk to the fetus, antenatal corticosteroids were immediately administered to promote lung maturation and magnesium sulfate was started for fetal neuroprotection. Subsequently, the decision was made to perform an atraumatic “en caul” cesarean delivery, as the safest option for an emergency delivery in a preterm pregnancy [1]. After successful “en caul” delivery, the MiniMizer ring, which had dislodged downwards and led to mesenteric ischemia, was revealed. Intraoperative esophagogastroduodenoscopy revealed a defect in the stomach wall, 1 cm-long and gastric-ring-related, covered with a layer of purulent exudate. Upon further exploration, a herniation of the distal alimentary loop through the Petersen foramen was observed. Successful management included ring removal and intestinal loop reduction from the Petersen’s space, without evidence of strangulation, what was confirmed with the indocyanine green (ICG) angiography. Partial resection of the bowel was not necessary; however the patient was scheduled for a second-look relaparotomy to reassess bowel viability.

**Fig. 2:**
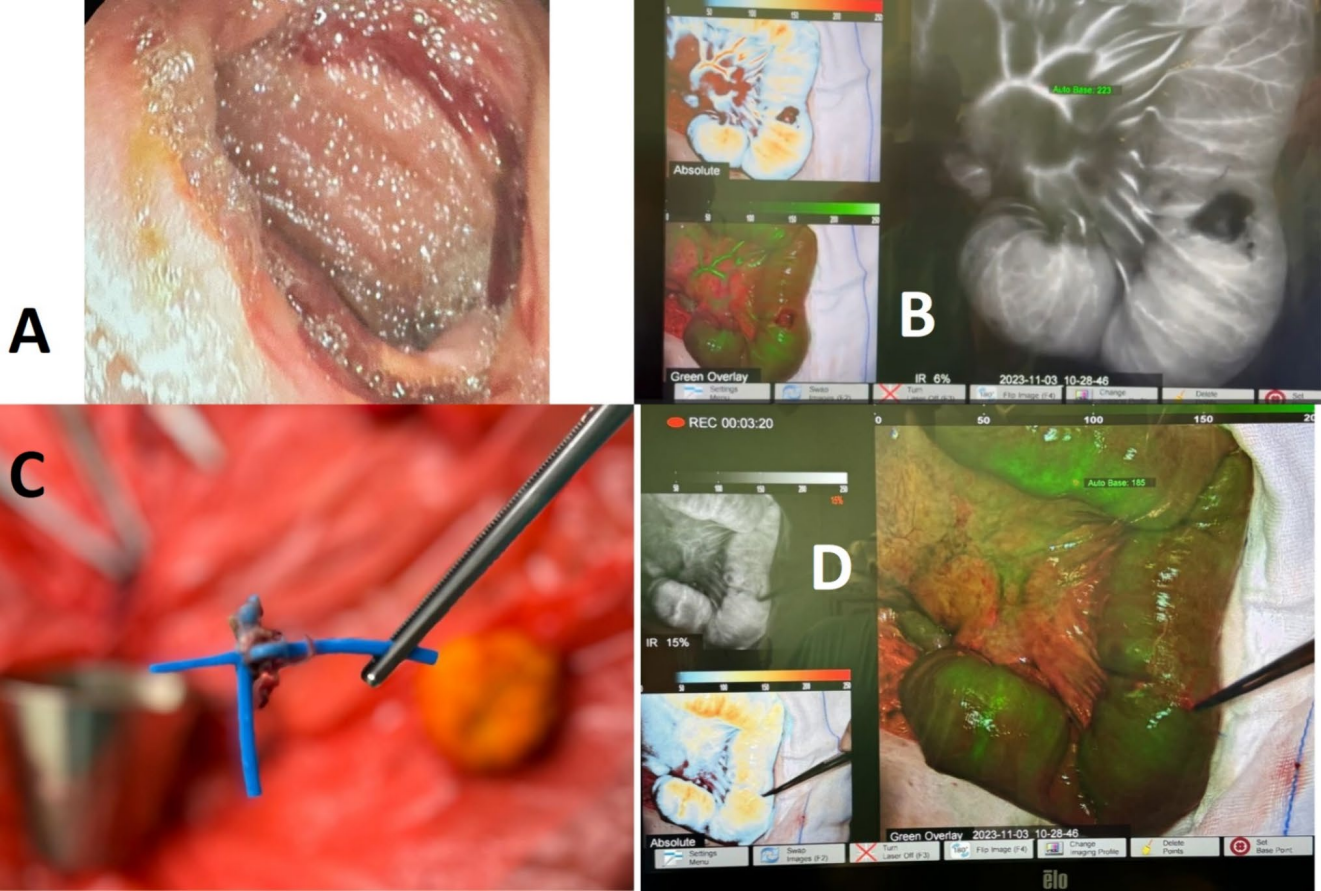
Selected images from the presented case. A – gastric-ring-related defect in the stomach wall showed by intraoperative esophagogastroduodenoscopy; B and D – use of indocyanine green (ICG) angiography to asses intestinal vascularization; C – removal of the Minimizer Ring

The following day, a second-look relaparotomy was performed. Proper intestinal peristalsis was observed, intestinal vascularization was reconfirmed using ICG angiography, and the Petersen’s space was closed medially to prevent future herniation. Selected images from the surgical procedure are presented in Fig. 2.

Total parenteral nutrition was administered to the mother until she met the requirements to receive food by the oral route. The postoperative course was uneventful.

## Discussion

Obesity, whose prevalence among women of childbearing age continues to rise, may be associated with numerous short- and long-term complications for both, the mother and the infant [2, 3]. Women with history of a bariatric surgery to manage obesity need to be informed that the probability of becoming pregnant after the procedure is increased [2]. Delayed conception – at least 12 to 18 months – after weight loss surgery is advised by various international guidelines [2, 3]. Still, the literature offers reports about various complications during pregnancy after a bariatric surgery (Table 1).

**Table 1:** Complications in pregnancy after bariatric surgery

Authors	Parameter							
	Age	BMI [kg/m^2^]	WG	Symptoms	Surgery	Time interval	Cause	Troubleshooting
Ongso [4]	27	25.9	6	colicky periumbilical abdominal pain, nausea	gastric band	4 years	tubing-related complication – adhesion band around the bowel	diagnostic LSC; band removal; adhesiolysis; delivery at term
Policiano [5]	37	19	33	abdominal pain, nausea, vomiting, belching, gastric regurgitation, solid and liquid intolerance; abnormal CTG of the fetus	gastric band	4 years	hemoperitoneum; gastric rupture above gastric band	enlargement of the gastric band; emergency laparotomy and cesarean section due to decelerations; band removal; partial gastrectomy
Didziokaite [6]	41	26.4	27	stabbing upper abdominal pain, radiating to the scapula	gastric band	13 years	acute peritonitis; 1 cm gastric-band-related defect in the stomach wall covered with purulent exudate	diagnostic LSC; intraoperative esophagogastroduodenoscopy; peritoneal washing of the abdominal cavity; the band remains
De Raaff [7]	30	n/d	33	epigastric pain with radiation to the back	1. LAGB, 2. minimizer ring	1.14 years 2.6 months	ileus; slippage of the ring	CT; diagnostic LSK; ring removal
Suffee [8]	29	n/d	30	3-day history of severe dysphagia	LAGB	4 years	slippage of the band	radiographic abdominal scout image; diagnostic LSC; band removal

Abbreviations: BMI – body mass index; CT – computed tomography; CTG – cardiotocography; LAGB – laparoscopic adjustable gastric banding; LSC – laparoscopy; time interval – time from surgery to pregnancy; WG – weeks of gestation; n/d – no data

Multivitamin and mineral supplementation during the preconception period and pregnancy should include the following: folic acid (4–5 mg throughout the first trimester), vitamin B12 (1 mg every 3 months during preconception by intramuscular depot injection and monthly during pregnancy), iron (min. dose of 45 mg elemental iron daily, > 18 mg for adjustable gastric band), thiamine (> 12 mg). Vitamin D should be supplemented to maintain a concentration of ≥ 50 nmol/L with serum parathyroid hormone within normal limits [2].

In the presented case, the patient became pregnant one month after the bariatric surgery and failed to comply with the supplementation recommendations. Oral glucose tolerance test was performed at 24 weeks of pregnancy, even though it is contraindicated, or at least highly controversial, after bariatric surgery [2]. Despite possible micronutrient deficiencies, the pregnancy was uneventful for the fetus, while the life of the patient was threatened by a complication related to gastric ring displacement.

Laparoscopic sleeve gastrectomy (LSG), Roux-en-Y gastric bypass (RYGB), banded Roux-en-Y gastric bypass, laparoscopic adjustable gastric banding (LAGB), and OAGB are currently the forefront surgical procedures in bariatric surgery [3]. Gastrojejunostomy stenosis, erosions, and ring migration (slippage) occur in up to 7% (range: 1.5–7%) of all banded bariatric procedures [3]. The MiniMizer ring-related complications can be either acute or chronic. Surgical exploration and band removal is the recommended emergency treatment of choice. In case of our patient, migration of the MiniMizer ring coincided with small bowel obstruction, mesenteric ischemia, and internal herniation of the small intestine.

## Conclusion

As far as bariatric surgery is concerned, the possibility of peri- and postoperative complications, which in time might lead to various maternal and fetal risks, should not be ignored. The case of our patient illustrates that and may serve as a cautionary tale, as maternal life was seriously threatened. Therefore, it is advised to inform the patient about the importance of maintaining sufficient interval of time between a bariatric surgery and conception.

Also, a growing use of indocyanine green angiography as a means of assessing intestinal viability, which can help prevent premature bowel resection, should be noted.

## Data Availability

No datasets were generated or analysed during the current study.
